# Autologous Bone Marrow Mesenchymal Stem Cells Associated with Tantalum Rod Implantation and Vascularized Iliac Grafting for the Treatment of End-Stage Osteonecrosis of the Femoral Head

**DOI:** 10.1155/2015/240506

**Published:** 2015-02-23

**Authors:** Dewei Zhao, Baoyi Liu, Benjie Wang, Lei Yang, Hui Xie, Shibo Huang, Yao Zhang, Xiaowei Wei

**Affiliations:** ^1^Department of Orthopedics, Zhongshan Hospital of Dalian University, Jiefang Street No. 6, Dalian, Liaoning 116001, China; ^2^Columbia Center of Translational Immunology, Columbia University College of Physicians and Surgeons, 650 West 168th Street, New York City, NY 10032, USA

## Abstract

Tantalum rod implantation with vascularized iliac grafting has been reported to be an effective method for the treatment of young patients with osteonecrosis of the femoral head (ONFH) to avert the need for total hip arthroplasty (THA). However, there have been unsatisfactory success rates for end-stage ONFH. The authors describe a modified technique using bone marrow mesenchymal stem cells (BMMSCs) associated with porous tantalum rod implantation combined with vascularized iliac grafting for the treatment of end-stage ONFH. A total of 24 patients (31 hips) with end-stage ONFH were treated with surgery; ARCO IIIc stage was observed in 19 hips and ARCO IV stage was observed in 12 hips. All patients were followed for a mean time of 64.35 ± 13.03 months (range 26–78). Operations on only five hips were converted to THA. The joint-preserving success rate of the entire group was 89.47% for ARCO stage IIIc and 75% for ARCO stage IV. The mean Harris hip score of the 31 hips improved significantly from 38.74 ± 5.88 points (range 22–50) to 77.23 ± 14.75 points (range 33–95). This intervention was safe and effective in delaying or avoiding total hip replacement for end-stage ONFH.

## 1. Introduction

The femoral head is the most vulnerable site for the development of osteonecrosis, and the incidence is increasing; every year 10,000–20,000 new cases are diagnosed in the USA [1–3]. Half of the patients requiring treatment for this problem are younger than 40 years [1, 4]. Five distinct conditions are known to have a high causal link with osteonecrosis of femoral head (ONFH): steroid use, alcohol abuse, trauma, coagulopathies, and abnormal vascular anatomy [1, 2, 5]. Despite improvements in hip arthroplasty design and techniques, it is unlikely that prosthetic replacements will endure for life [6]. A variety of surgical procedures have been attempted to maintain the femoral head and avoid total hip arthroplasty (THA) in younger patients; these approaches include core decompression [7, 8], various types of osteotomies [9], and nonvascularized bone grafting [8]. However, none of these therapeutic techniques are consistently reliable. Commonly, patients have few treatment choices other than joint arthroplasty when subchondral collapse of the femoral head occurs.

Vascularized bone flap transfer is effective for the treatment of femoral head osteonecrosis [10–17]; however, vascularized bone grafting leads to excellent results in hips in the early stages of the disease. Once the head has collapsed, the results are less predictable [18]. Developing approaches to prevent femoral head collapse in young patients and to avoid premature hip arthroplasty has attracted increasing attention. Several reports have demonstrated the valuable role of porous tantalum rods in ONFH treatment [19–22]. Porous tantalum has a high volumetric porosity, and its modular elasticity is similar to subchondral bone; however, its strength and endurance limit are better than natural bone grafts [23]. Based on these biological characteristics of porous tantalum, the authors developed a technique that uses porous tantalum rod implantation combined with vascularized iliac grafting for the treatment of ONFH [24]. However, that report described an unsatisfactory success rate for ARCO stage IV ONFH. Based on the failed cases, the authors determined that there was no bone growth into the top of the tantalum rod.

Some pioneering studies have demonstrated the efficacy of autologous bone marrow cell implantation into the femoral head for early-stage ONFH [25–27]. In such procedures, several tens of thousands of bone marrow stem cells, which were isolated and concentrated from anterior iliac crest-aspirated bone marrow, were implanted into the osteonecrotic zone of the femoral head right after CD. The efficacy and safety of this novel protocol of autologous implantation of* ex vivo* expanded bone marrow mesenchymal stem cells (BMSCs) into the femoral head for the treatment of early-stage ONFH has been demonstrated [28]. Based on the role of BMSCs, the authors developed a modified technique using BMSCs associated with porous tantalum rod implantation combined with vascularized iliac grafting for the treatment of end-stage ONFH.

This paper describes the outcomes of the modified technique for the treatment of advanced ONFH. The authors retrospectively reviewed 24 patients (31 hips) and evaluated the clinical and radiographic outcomes. All patients were informed that data from their clinical and radiological examinations would be submitted for publication.

## 2. Materials and Methods

This single-center randomized clinical trial was conducted in a university-affiliated hospital in China between October 2007 and October 2008. The objective of this study was to assess the efficacy of a protocol involving BMSCs associated with tantalum rod implantation combined with vascularized iliac grafting for the treatment of end-stage ONFH. The protocol of the present study was approved by the Institutional Review Board of Dalian University and the Ethics Committee of the City of Dalian under the authorization of the Ministry of Public Health of China. Written informed consent was obtained from each patient before enrollment.

Patients were evaluated both clinically and radiologically using the Association Research Circulation Osseous (ARCO) classification [29] and the Harris hip score (HHS) [30] preoperatively and at the end of the follow-up. We recommend the following criteria for selecting patients for this procedure: (1) ARCO stage IIIc to IV disease, (2) femoral head collapse of >4 mm, and (3) no evidence of damage to the acetabular cartilage. Twenty-four patients (31 hips) were selected for this study. The patients included 13 males and 11 females with a mean age of 33.21 ± 6.09 years old (range 23–45) at surgery. The etiology of the osteonecrosis was corticosteroid use in 14 hips, alcohol abuse in 4 hips, a traumatic event in 2 hips, and idiopathic or unknown in 4 hips; 19 hips had stage IIIc osteonecrosis and 12 had stage IV osteonecrosis. The mean preoperative Harris hip score was 36.29 ± 6.11 points (Table 1). The outcomes were evaluated based on the HHS and the radiological results. Surgical time and blood loss were recorded.

The tantalum rod (Trabecular Metal Technology; Zimmer, Inc., Warsaw, Indiana) is made entirely of porous tantalum and has a 10 mm diameter cylindrical shape, lengths from 70 to 130 mm available in 5 mm increments, a threaded section designed to engage the lateral cortex of the femur, and a hemispherical tip for supporting the subchondral plate.

### 2.1. Culture and Expansion of BMMSCs

Bone marrow aspiration was performed in all patients two weeks before surgery [28, 31]. The 10 mL of bone marrow recovered from the posterior superior iliac spine was suspended in 100 mL of phosphate-buffered saline (PBS) solution containing 10% autologous serum and 100 U/mL of heparin to avoid clotting. The mononuclear cells were isolated using Ficoll density gradient centrifugation and were washed with PBS containing 10% homologous serum; they were then resuspended in minimum essential medium alpha (alpha-MEM) containing 10% autologous serum at a concentration of 10^5^ cells/mL. A small aliquot of cells was used for immunofluorescence identification. Then, the cells were seeded into 50 mL cell culture flasks and incubated in 5% CO_2_ at 37°C. After one day, the nonadherent cells were discarded, and fresh alpha-MEM containing 10% autologous serum, 100 U/mL penicillin, 100 mg/mL streptomycin, and 2 mM L-glutamine was added to the flask. At approximately 80% confluence, the cells were resuspended with trypsin-EDTA buffer, washed, and resuspended at 10^4^ cells/mL with alpha-MEM containing 10% autologous serum, 100 U/mL penicillin, 100 mg/mL streptomycin, and 2 mM L-glutamine, before being seeded into new flasks. When the cells reached confluence, the culture medium was removed, and the cells were trypsinized and washed before being suspended in normal saline at a concentration of 10^6^ cells/mL × 2 mL for transplantation. The entire BMMSCs culture and expansion period was approximately two weeks.

### 2.2. Immunofluorescence Microscope

Each small aliquot of cells was cultured in a 24-well plate for 5 days. Then, the cells were washed in phosphate-buffered saline (PBS, Sigma-Aldrich, Milwaukee, WI, USA) and fixed in a 4% paraformaldehyde solution (Sigma-Aldrich, Milwaukee, WI, USA) for 10 min; 3% peroxidase eliminated endogenous hydrogen peroxide; and CD105, CD34, CD45 (1 : 10) (BD Biosciences, San Jose, CA) with fluorescent antibody were added after extensive washing with PBS. Cells were incubated in 5% CO_2_ at 37°C for two hours. Cells were mounted in glycerol/buffer on a glass slide after extensive washing with PBS. Images of the labeled cells were obtained using a fluorescence image restoration microscope (Applied Precision, USA).

### 2.3. Surgical Technique

Under general anesthesia, each patient was placed in the supine position, with the affected hip elevated by 30°. In the lateral hip region, a double “S-” shaped incision measuring 8–12 cm in length was made, starting from 1 cm lateral to the anterior superior iliac spine and extending to the greater trochanter. An approximately 3.5 cm × 2 cm vascularized bone graft with the inner plate of the ilium, pedicled with the ascending branch of the lateral femoral circumflex vessels, was harvested from the anterosuperior iliac crest. The pedicled bone block was placed in saline-moistened gauze; cancellous iliac bone chips were harvested and cocultured with the BMMSCs for later transplantation [15, 24].

The hip joint was approached through the space between the sartorius and the tensor fasciae latae superficially and between the gluteus medius and the rectus femoris inferiorly. The anterior aspect of the joint capsule was excised to expose the border of the femoral head and neck. Any osteophytes were excised because they restrain the hip range of motion. The necrotic lesion was excised and curetted through a 3 × 2 cm bone window created at the anterior aspect of the femoral head-neck junction. A high-speed abrasive drill was used to remove the femoral head lesion as completely as possible until bleeding was observed [15, 24].

**Figure 1:**
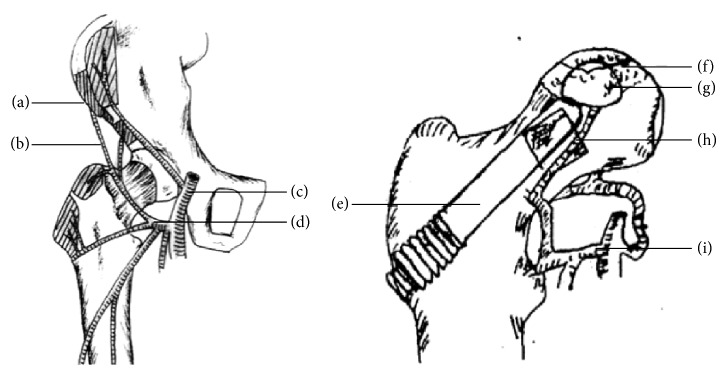
Illustration of the surgical anatomy. ((a) and (g)) Vascularized bone graft from anterosuperior iliac crest. ((b) and (h)) The ascending branch of lateral femoral circumflex vessels. (c) The deep femoral artery. ((d) and (i)) Lateral femoral circumflex artery. (e) Tantalum rod. (f) Cancellous iliac bone chips with BMMSCs in necrosis zone.

A tantalum rod implantation procedure similar to core decompression was completed. A guide wire was drilled from the lateral femoral cortex directly to the original lesion area in the femoral head with C-arm radiographic guidance. A bone channel measuring 10 mm in diameter was created using a core reamer over the guiding needle. BMMSCs with cancellous bone chips harvested from the iliac crest were placed in the excavated region of the femoral head and impacted to elevate the collapsed segment of the femoral head. The vascularized iliac bone graft was modified to a hemispherical tip and then inserted and impacted obliquely into this area. Because the hemispherical construction could increase the stress area and to prevent stress concentration, care was taken to not squeeze the soft tissue cuff containing the vessels to the bone graft during insertion. The bone graft transplant was completed by exerting some pressure with the impaction instruments. A suitable tantalum rod was selected by fitting analogs to the channel and was inserted into the bony channel with its tip impacted into the cortical side of the bone graft (Figure 1). Last, the hip was moved slightly to confirm that the bone block would not be displaced, and the wound was closed in layers. The same surgeon (DWZ) performed all of the operations.

Patients were instructed to be non-weight-bearing for 4 weeks and partial weight-bearing for the subsequent 6 weeks. Full weight-bearing was achieved 6 months postoperatively [24].

### 2.4. Evaluation and Statistical Analysis

The evaluations consisted of clinical and radiographic analysis preoperatively and at the end of follow-up based on the HHS and ARCO staging. All patients underwent clinical and radiographic examinations in the third and sixth months postoperatively and then at 6-month intervals thereafter. A Kaplan-Meier survival curve was used to illustrate the failure of this treatment [32]. Failure and endpoints were defined as conversion to a THA. The survival rate was determined, and the success rates at both stages were compared. An excellent or good Harris hip score was defined as 80 or more points and a bad score was less than 70. The last follow-up was determined based on the time to conversion to THA or the longest hip survival duration. All analyses were completed using SPSS (Statistical Package for the Social Sciences) version 22.0 software (IBM SPSS Corp, New York, NY).

## 3. Results

### 3.1. Cells

**Figure 2:**
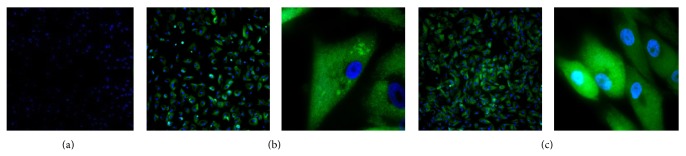
The results of cell surface antigen detection showed that BMSCs were hypso-expression CD44 and CD105, which are the identification marker of mesenchymal cell surface antigen. Meanwhile, BMSCs were hypoexpression CD34 which is the identification marker of hematopoietic cell surface antigen. (a) CD34 negative (×100), (b) CD44 positive, and (c) CD105 positive (×100, ×1000).

Cells grew on the bottom of the flask after 24 hours. They showed the characteristics of the bone marrow mesenchymal stem cells including a spindle shape, a swirl, and radial alignment. Immunofluorescence microscopy showed that they were CD45 and CD105 positive and CD34 negative (Figure 2).

### 3.2. Patients

There were 31 successful surgical procedures, with a mean operative time of 50 min and an average blood loss of 150 mL. All patients were followed for a mean of 64.35 ± 13.03 (26–78) months, without loosening of the internal fixation, postoperative infection, fractures, or other complications. Conversion to THA was performed in 4 patients (5 hips); ONFH in these cases was caused by corticosteroid use in 2 patients (3 hips), alcohol abuse in 1 hip, and idiopathic or unknown reasons in 1 hip.

### 3.3. Survival Rate

**Figure 3:**
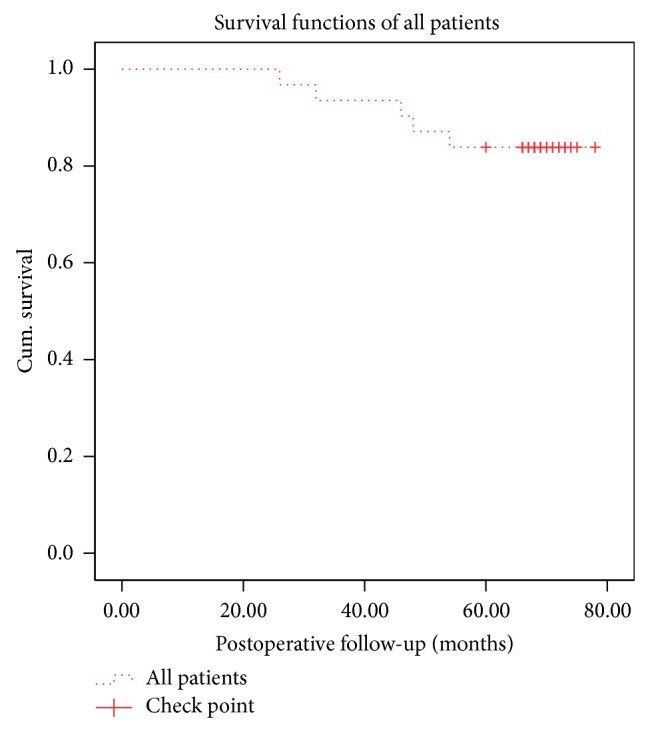
Kaplan-Meier survivorship curve with conversion to total hip arthroplasty as the end point. We set up the time point from 0 month to 80 months postoperatively on the *x*-axis. The *y*-axis shows the survival rate. The dotted line shows the survival rate for all of the hips. The survival rate of the entire group was 83.87%. The result shows that the applied protocol can be used for end-stage ONFH.

**Figure 4:**
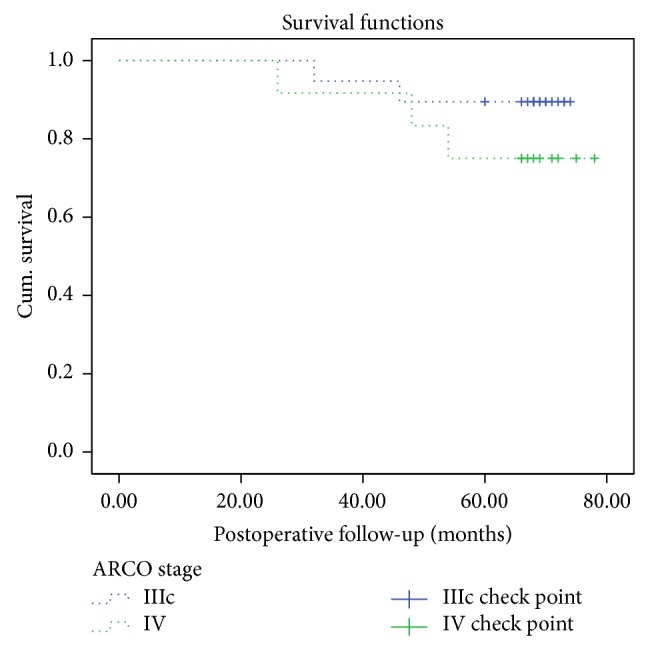
To understand how our procedure outcome is after operation, we made a Kaplan-Meier survivorship curve with conversion to total hip arthroplasty as the end point. We set up the time point from 0 month to 80 months on the *x*-axis. The *y*-axis shows the survival rate. The blue longer-dotted line shows the survival rate of stage III hips while the green shorter-dotted line shows the survival rate for ARCO stage IV hips. Looking across all of the hips we can see that the joint-preserving success rate of the stage IIIc group was 89.47% 5 years postoperatively while 75% for IV hips and the performance of total hip arthroplasty may emerge during the postoperative 20 months to 60 months. Now we have demonstrated the effect of this technique for end-stage osteonecrosis of the femoral head. However, comparison with a larger group or a control group of patients is necessary to further confirm the effect of this technique.

Five hips were converted to THA by the last follow-up including 2 hips with ARCO stage IIIc disease and 3 hips with ARCO stage IV disease (Figure 3). The average time between the joint-preserving surgery and conversion to THA was 41.20 ± 11.71 months (range, 26–54). The joint-preserving success rate of the entire group was 83.87% 5 years postoperatively, with 89.47% for stage IIIc disease and 75% for stage IV disease (Figure 4). The success rate for stage IV disease was lower compared to the success rate for stage III disease.

### 3.4. Harris Hip Score

The mean preoperative Harris hip score for all hips was 38.74 ± 5.88 (24–50) points, and the mean postoperative score was 77.23 ± 14.75 (33–95) points at last follow-up or before conversion to THA. The mean score for the survival hips improved from 39.04 ± 5.80 to 82.62 ± 7.65 points, with an advancement from 39.84 ± 4.86 to 79.11 ± 11.64 points for hips with stage IIIc osteonecrosis and from 37.00 ± 7.08 to 74.25 ± 18.86 points for hips with stage IV osteonecrosis (Table 2). Of the survival hips, 19 (73.1%) had excellent or good scores (scores of >80 points).

### 3.5. Radiographic Evaluation

**Figure 5:**
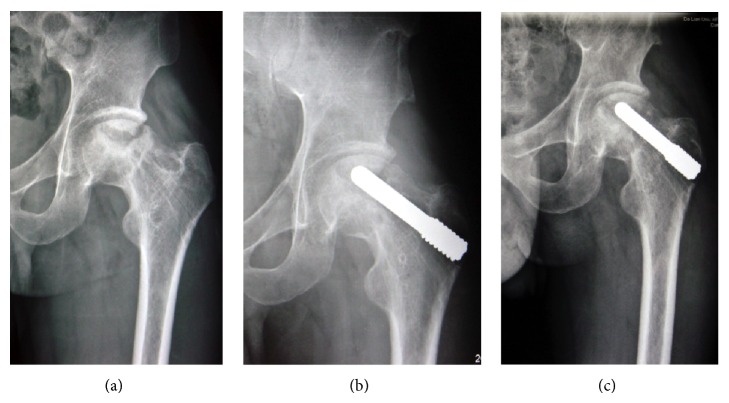
A 36-year-old male patient with bilateral ARCO stage III ONFH received the treatment of BMSCs associating with porous tantalum rod implantation combined with vascularized iliac grafting. Radiographs were taken before operation (a), 36 months after operation (b), and 60 months after operation (c). (c) The joint space remains preserved; the iliac bone graft is well incorporated.

**Figure 6:**
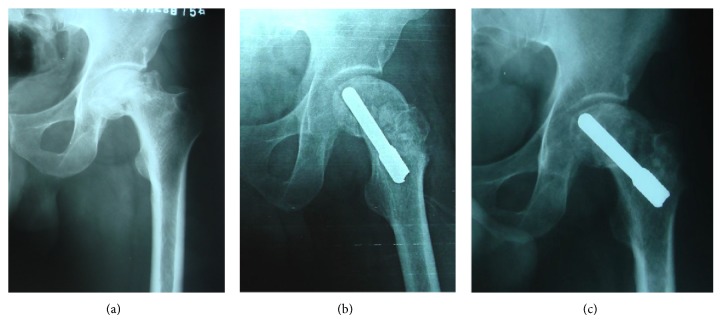
A 40-year-old male patient with ARCO stage IV ONFH at right received the treatment of BMSCs associating with porous tantalum rod implantation combined with vascularized iliac grafting. Radiographs were taken before operation (a), 18 months after operation (b), and 54 months after operation (c). (c) The femoral head remained circular, but collapse was found again in the femoral head.

Radiographic progression was observed in 3 of the 19 stage IIIc osteonecrosis hips. Two of these 3 hips were converted to THA. Sixteen (84.21%) of the 19 stage IIIc hips exhibited stable stage IIIc disease (Figure 5), and 7 hips had no radiographic progression of stage IV osteonecrosis (Figure 6).

## 4. Discussion

Osteonecrosis of the femoral head constitutes a multifactorial disease that mainly affects young adults [1, 2, 5, 8, 10, 14–16, 18]. The natural history of the clinical symptoms includes pain, dysfunction of the hip, and increasing patient disability. Fracture of the subchondral bone, articular cartilage collapse, and finally osteoarthosis of the hip joint are radiographic features that characterize the clinical progression of this disease [1, 2, 18]. Osteonecrosis of the femoral head progresses to femoral head collapse and articular incongruity in up to 70–80% of cases [33]. Total hip arthroplasty provides pain relief and restores function to millions of older patients with end-stage degenerative joint disease [6, 18, 34]. Mont et al. [35] reviewed all 48 patients (69 hips) younger than 30 years at the time of surgery who underwent a primary cemented THA. Eight hips were revised (three for infection and five for aseptic loosening), and one hip was dislocated and required open reduction. One additional cup was considered a radiographic failure. The 10-year survival was 83% with revision for any reason as the end point, and the 10-year survival was 90% with revision for aseptic loosening. However, there are many future challenges to the use of THA, particularly in younger and more active patients, in the future because of the lifetime of joint components [6].

Therefore, joint preservation is still the primary goal of treatment for young or active patients with ONFH. Conceptually, the ideal operation for this problem is one that removes the necrotic bone from the femoral head and replaces it with viable and structurally sound bone, thus restoring vitality to the femoral head and preventing collapse of the articular surface. Many joint-preserving surgeries have been performed, such as core decompression [7, 8, 33] and nonvascularized [8, 35] or vascularized bone grafting [11–17] with some successful results. However, a decision-making hierarchy, based on radiographic findings and the philosophy of performing the least invasive treatment appropriate for the extent of the disease, was presented for the treatment of patients with ONFH. Vascularized bone grafting alone was recommended for the treatment of stage III disease as joint-preserving surgery in that decision-making hierarchy [18]. The rationale for vascularized bone grafting is that it allows decompression, provides structural support, and restores a vascular supply that had been deficient or nonexistent for a long period of time. There have been multiple published reports on the use of vascularized fibular and iliac grafts [10, 12, 14–16].

Berend et al. [36] analyzed 224 collapsed osteonecrotic hips (in 188 patients) treated with vascularized fibular grafts and found a survival rate of 64.5% at a mean of 4.3 years (range, two to twelve years). Marciniak et al. [12] reported clinical data including treatment for 23 Marcus-Enneking stage III hips and 64 stage IV hips with vascularized fibular grafting; 16 (70%) stage III hips and 38 (59%) stage IV hips survived for at least five years. Our research team [15] retrospectively reviewed 197 patients (226 hips) treated with iliac bone block transfer perfused by the ascending branch of the lateral femoral circumflex artery; successful results were achieved in 96% of the patients with Ficat and Arlet stage II osteonecrosis, 90% with stage III osteonecrosis, but 57%, which is a low success rate, with stage IV osteonecrosis. In summary, vascularized bone grafting can lead to excellent results in hips with early-stage disease. The procedure may be effective for larger lesions just before head collapse. Once the head has collapsed, the results are less predictable.

In our study, we followed a group of 24 patients (31 hips) with end-stage osteonecrosis of the femoral head who were treated with bone marrow mesenchymal stem cells (BMSCs) associated with porous tantalum rod implantation combined with vascularized iliac grafting over an average of 64.35 ± 13.03 months, and the joint-preserving success rate of the entire group was 89.47% for ARCO stage IIIc and 83.33% for ARCO stage IV. The mean Harris hip score of the 31 hips improved significantly from 36.29 ± 6.11 (22–47) points to 77.23 ± 14.75 (33–95) points. This research shows a promising outcome for end-stage ONFH. The success rate of our procedure was significantly higher than that of single vascularized iliac grafting for end-stage ONFH [15].

We maintain that the difference of outcomes was due to the role of porous tantalum and bone marrow mesenchymal stem cells. Porous tantalum has been developed as a biomaterial for a variety of surgical applications, such as vascular clamps, and as a bone graft substitute. It is also called trabecular metal due to its mechanical strength, porosity, and excellent biocompatibility for use* in vivo* [37]. It has a high volumetric porosity, and its modular elasticity is similar to subchondral bone, but its strength and endurance limit are better than those of natural bone grafts. Mesenchymal stem cells are pluripotent cells found in multiple human adult tissues including bone marrow, synovial tissues, and adipose tissues. Bone marrow mesenchymal stem cells have been shown to differentiate into bone, cartilage, muscle, and adipose tissue [38–40].

In our previous study [28], approximately 10 mL of bone marrow from the subtrochanteric region was directly aspirated once the decompression tunnel was established during the surgery, avoiding the need for bone marrow aspiration from the iliac crest. The expansion of autologous BMMSCs* ex vivo* allowed the harvesting of sufficient amounts of cultured BMMSCs within two weeks and the preparation of approximately 2 × 10^6^ BMMSCs for femoral head implantation. We found that obtaining a significantly larger number of BMMSCs through* ex vivo* expansion of autologous BMMSCs from a relatively small volume of subtrochanteric bone marrow aspirated through the core decompression tunnel is a safe, reliable, and highly effective procedure. The porous tantalum replaced the necrosis bone with viable and structurally sound bone; then, the autologous bone marrow cell implantation restored vitality to the femoral head in our surgical protocol. Perhaps, this is the major reason for the better outcomes of treatment for end-stage ONFH with autologous bone marrow cells with porous tantalum rod implantation and vascularized iliac grafting compared to other vascularized bone graft procedures. However, the previous results with porous tantalum rods or autologous bone marrow cell implantation without vascularized bone grafting for postcollapse stage ONFH were unsatisfactory.

Nadeau et al. [21] evaluated the short term clinical outcomes of a porous tantalum implant for the treatment of 15 patients with 18 osteonecrotic hips with Steinberg stage III (3 hips) and IV (15 hips) disease. The success rate 12 months postoperatively was 77.8%, and the overall success rate was 44.5%. Hernigou and Beaujean [25] followed 189 hips in 116 patients for five to ten years after core decompression and grafting combined with implantation of autologous bone marrow obtained from the iliac crest. Success (avoidance of total hip replacement) was achieved in 136 (94%) of 145 hips that had been operated on before femoral head collapse but in only nineteen (43%) of forty-four hips that had been operated on after collapse.

Furthermore, restoring the blood supply was a very important factor for joint-preserving procedures. The blood supply restoration was evaluated based on hemodynamic changes after treatment of ONFH with vascularized iliac grafting using DSA in our previous research [41, 42]. The iliac graft pedicled with the ascending branch of the lateral femoral circumflex vessels can provide blood supply to the femoral head, similar to a free vascularized fibular graft. In addition, the location and role of the vascularized iliac grafting were the same as those of the most important vessel for the femoral head, the superior retinacular artery [43]. This is because the vascularized iliac bone graft was inserted into the necrotic lesion from the bone window in the anterior aspect of the femoral head-neck junction where the entry point of the superior retinacular artery is located [44]. Removal of the necrotic bone could restore blood perfusion of the necrosis zone because of the elimination of the “hard zone.” The extent to which the removal of necrotic bone is complete determines the efficacy of the surgery [43]. In this surgical procedure, a high-speed abrasive drill was used to remove the lesion in the femoral head as completely as possible until bleeding was observed. However, completely removing the necrotic bone decreased the stability of the femoral head and increased the risk of radiographic progression. This is the major reason that radiographic progression was observed in 8 hips (25.81%) including 3 hips with stage IIIc disease and 5 hips with stage IV disease. Therefore, extended bed rest and non-weight-bearing during the early postoperative period were necessary because of the weak structural integrity of the bone graft.

The success of the procedure is related to multiple factors: (1) decompression of the femoral head, which may abolish the ischemia due to increased intraosseous pressure; (2) excision of the necrotic bone underneath the weight-bearing region that might inhibit revascularization of the femoral head; (3) buttressing of the articular surface with the vascularized fibular graft through primary callus formation augmented by additional cancellous bone grafts, which has osteoinductive and osteoconductive factors; and (4) protection of the healing structure with a period of limited weight-bearing [10]. The surgical protocol involving autologous bone marrow cells with tantalum rod implantation and vascularized iliac grafting was designed based on all these factors. The joint-preserving success rate of the entire group was 83.87% 5 years postoperatively, with 89.47% for stage IIIc disease and 75% for stage IV disease in the current study. The applied protocol was useful for the joint-preserving procedure in end-stage ONFH.

## 5. Conclusions

The current results show that autologous bone marrow cells with tantalum rod implantation and vascularized iliac grafting can be used for end-stage osteonecrosis of the femoral head. However, comparison with a larger group or a control group of patients is necessary to further confirm the effect of this technique.

## Figures and Tables

**Table 1:** Patient demographics.

Gender	Number of patients

Males	13
Females	11

Invasive hip	Number of patients

Unilateral	17
Bilateral	7

Etiology	Number of patients

Idiopathic	4
Corticosteroids	14
Alcohol	4
Trauma	2

ACRO staging	Number of hips

Stage IIIc	19
Stage IV	12

**Table 2:** Clinical results of survival hips.

ARCO stage	Survival *N*	HHS (preoperative)	HHS (postoperative)	EGR *N*, %
IIIc	17	39.84 ± 4.86	79.11 ± 11.64	12, 63.16%
IV	9	37.00 ± 7.08	74.25 ± 18.86	7, 58.33%

ARCO, Association Research Circulation Osseous; HHS, Harris hip score. EGR means excellent and good rate.
